# A Near-Wall Methodology for Large-Eddy Simulation Based on Dynamic Hybrid RANS-LES

**DOI:** 10.3390/e26121095

**Published:** 2024-12-14

**Authors:** Michael Tullis, D. Keith Walters

**Affiliations:** Department of Mechanical Engineering, University of Arkansas, Fayetteville, AR 72701, USA; mrtullis@uark.edu

**Keywords:** computational fluid dynamics, boundary layer, turbulence, large-eddy simulation, wall modeling

## Abstract

Attempts to mitigate the computational cost of fully resolved large-eddy simulation (LES) in the near-wall region include both the hybrid Reynolds-averaged Navier–Stokes/LES (HRL) and wall-modeled LES (WMLES) approaches. This paper presents an LES wall treatment method that combines key attributes of the two, in which the boundary layer mesh is sized in the streamwise and spanwise directions comparable to WMLES, and the wall-normal mesh is comparable to a RANS simulation without wall functions. A mixing length model is used to prescribe an eddy viscosity in the near-wall region, with the mixing length scale limited based on local mesh size. The RANS and LES regions are smoothly blended using the dynamic hybrid RANS-LES (DHRL) framework. The results are presented for the turbulent channel flow at two Reynolds numbers, and comparison to the DNS results shows that the mean and fluctuating quantities are reasonably well predicted with no apparent log-layer mismatch. A detailed near-wall meshing strategy for the proposed method is presented, and estimates indicate that it can be implemented with approximately twice the number of grid points as traditional WMLES, while avoiding the difficulties associated with analytical or numerical wall functions and modified wall boundary conditions.

## 1. Introduction

The scale-resolving methods for computational fluid dynamics simulations have become increasingly viable for practical application to realistic science and engineering problems. Compared to Reynolds-averaged Navier–Stokes (RANS) turbulence modeling, methods such as large-eddy simulation (LES), detached-eddy simulation (DES), and hybrid RANS-LES offer potentially significantly more accuracy at the cost of additional computational resources, including memory and processing time/power. This is because some or all of the large-scale, energy-containing eddies primarily responsible for the turbulent transport of momentum and heat are directly resolved in the simulations rather than statistically represented using empirically derived model equations. For free shear flows, the largest turbulent eddies tend to scale with the thickness of the shear layer and can often be well represented on meshes with resolution comparable to that which is required to represent the scales of the mean (Reynolds-averaged) flow field. For wall-bounded flows, the mesh resolution required to resolve even the largest turbulent eddies throughout the entire boundary layer is substantially greater than that which is required to resolve the mean flow, especially in the wall-parallel directions, and the difference increases with the increasing Reynolds number [1]. Together with the time step restrictions needed to resolve the corresponding temporal scales, this makes the near-wall region in wall-bounded turbulent flow an ongoing challenge for the practical simulation of complex flows using LES.

For turbulent wall-bounded flow, the primary energy-containing eddies in the buffer layer, where turbulent kinetic energy and its production reaches a peak, scale with the viscous length scale ($\delta_{\nu }$) dictated by the friction velocity, $u_{\tau }$, and the fluid kinematic viscosity, ν:

(1) $$\delta_{\nu }\equiv \frac{\nu }{u_{\tau }},$$

(2) $$u_{\tau }\equiv \sqrt{\frac{\tau_{w}}{\rho }},$$

where $\tau_{w}$ and ρ are the local wall shear stress and fluid density [1]. The largest turbulent eddies in the boundary layer, which are primarily responsible for mixing in the outer layer, scale with the boundary layer height, δ. As the Reynolds number increases, the ratio $\delta_{\nu }/\delta$ decreases, and full resolution of the large-scale eddies within the boundary layer requires higher mesh resolution levels. Since turbulent eddies are highly three-dimensional, this increased resolution is required in all three coordinate directions. As a consequence, the number of mesh points N required for full wall-resolved large-eddy simulation (WRLES) scales as [2]:

(3) $$N\;\sim \;{Re}^{13/7}.$$

This requirement places a significant constraint on the feasibility of WRLES for wall-bounded turbulent flows, especially for applications at a high Reynolds number.

One approach to address this is to use hybrid RANS-LES modeling. The standard approach for hybrid RANS-LES is to apply a RANS model in the attached boundary layer regions and transition the simulation to an LES or LES-like method in separated or free shear flow regions. The first model to effectively implement such an approach for general three-dimensional flows was the detached-eddy simulation (DES) [3]. The challenge for hybrid modeling is effectively transitioning between two mathematically distinct equation forms (filtered versus Reynolds-averaged) in a physically appropriate manner. As such, research has often focused on addressing such well-known problems as log-layer mismatch, delayed shear layer breakdown, and the shielding of the boundary layer to avoid the premature introduction of resolved turbulence fluctuations. Subsequent model versions of DES have been proposed and found to improve predictive capability by effectively addressing these issues [4,5]. Several other hybrid RANS-LES model forms have been proposed, and reviews are available in Refs. [6,7].

Bhushan and Walters [8] proposed a general dynamic hybrid RANS-LES (DHRL) framework to address these challenges. The method is based on matching turbulent kinetic energy production in the transition zone between RANS and LES, and it is agnostic to the choice of the RANS model and the LES subgrid stress model. Results have shown that DHRL can be used either in the standard hybrid modeling approach, in which the boundary layer is primarily simulated using RANS, or in a manner that allows substantial resolution of turbulent energy content within the boundary layer. Which approach is adopted by the model is dictated by the mesh resolution level and discretization scheme for a particular problem [9]. To date, the DHRL framework has primarily been used with a combination of the k−ω SST eddy viscosity RANS model component and monotonicity-preserving implicit large-eddy simulation (MILES) [10,11,12,13,14].

A second approach that is often adopted to mitigate the stringent near-wall mesh requirements for WRLES is wall-modeled large-eddy simulation (WMLES), in which the grid is constructed to resolve the large eddies in the outer layer of the boundary layer, i.e., those which scale with boundary layer height δ, and a modified near-wall treatment is used to properly couple the wall shear stress and the resolved velocity field. The simplest WMLES formulations use algebraic wall functions that prescribe the dimensionless near-wall velocity distribution as a function of the wall shear stress and wall-normal distance, d. The earliest wall functions were based on the well-known log-layer distribution:

(4) $$u^{+}=\frac{1}{\kappa }\ln y^{+}+B,$$

where $y^{+}$ and $u^{+}$ are the inner-scaled dimensionless wall distance and velocity:

(5) $$y^{+}\equiv \frac{u_{\tau }d}{\nu },$$

(6) $$u^{+}\equiv \;\frac{u}{u_{\tau }},\;$$

κ is the von Karman constant, and B is an empirically determined universal constant [15]. More advanced algebraic functions include prescription of the velocity profile in the buffer region and viscous sublayer [16] and/or the effect of a pressure gradient [17].

An alternative to algebraically prescribed wall functions is the numerical solution of locally one-dimensional governing equations for wall-parallel velocity components based on the thin boundary layer equations (TBLEs). Turbulent stress terms are modeled using eddy viscosity and a simple algebraic closure such as a mixing length model [18,19]. The TBLE approach eliminates assumptions regarding the shape of the near-field velocity profile, allowing for a more natural response to pressure gradients, although the use of locally 1D approximation results in an error in the solution relative to full WRLES. It has also been shown that the inclusion of nonlinear convective terms in the TBLEs can result in the manifestation of a resolved Reynolds stress term in the TBLE region, which must be accounted for using methods such as the modification of the incorporated mixing length model [20,21].

Several other attempts to blend the LES and RANS models to effectively model the near-wall region can be found in the open literature. Many of these have proposed a similar approach to that which is used in the current study, in which a single three-dimensional mesh is used for the simulations, with sufficient refinement in the wall-normal direction to allow for the resolution of the mean flow and the modification of the modeled stress terms to compensate for the lack of refinement in the streamwise and spanwise directions. Examples include the use of precomputed DNS data from a simple channel flow test case to inform the near-wall eddy viscosity [22], and the blending of the LES simulation with a one-equation RANS model in the near-wall region based on dimensionless wall distance [23]. The constrained large-eddy simulation approach uses a RANS eddy viscosity to modify the LES subgrid stress close to the wall, with an a priori interface selected to delineate the inner and outer regions [24]. A more recently proposed method [25] demarcates the flow into three zones, including the freestream and near-wall regions, as well as a hybrid region sandwiched between the two. The LES and RANS equations are solved simultaneously, and the eddy viscosity used in the momentum equations is obtained via interpolation between the RANS value and the effective LES value. Interested readers are referred to Refs. [6,26] for a more complete review of the wall-modeling techniques in LES.

The objective of the study presented in this paper is to perform initial validation tests for a proposed wall treatment method for LES that avoids the use of either algebraic wall functions or one-dimensional numerical treatments based on a TBLE. The concept is based on blending the simulation between effectively RANS in the very-near-wall region to effectively LES in the outer region. The mesh is therefore constructed with streamwise and spanwise resolutions equivalent to a typical WMLES. The mesh resolution in the wall-normal direction is also equivalent to WMLES in the outer layer but adopts stretching in the near-wall region to produce a mesh comparable to that used for a typical RANS simulation. Mesh guidelines are developed and presented in order to compare the overall mesh count and computational expense with both the WRLES and WMLES methods. Simulation results are obtained for a canonical channel flow test case at two different Reynolds numbers and compared to DNS results available in the open literature.

## 2. Materials and Methods

In the proposed wall-modeling approach, hereafter referred to in this paper as wall mean-resolved LES (WMRLES), the boundary layer mesh is constructed such that pure LES is supported in the outer layer and hybrid RANS-LES is supported in the inner near-wall layer. The streamwise and spanwise mesh sizing is therefore equivalent to that used in WMLES. The wall-normal mesh sizing is also equivalent to WMLES in the outer layer but equivalent to RANS in the inner layer. This approach eliminates the need for analytical or numerical wall functions to be applied at the wall boundary, at the expense of additional mesh resolution in the near-wall layer. In the present approach, the mean flow near the wall is solved using the full three-dimensional form of the momentum equation and is integrated directly with the LES simulation, both numerically and in terms of the mesh. The key aspects of the method are the dynamic hybrid RANS-LES (DHRL) framework used to blend RANS and LES modeling in the near-wall region, and the mixing length model used to specify the near-wall RANS modeling component of DHRL.

### 2.1. Near-Wall Modeling Methodology

#### 2.1.1. Dynamic Hybrid RANS-LES Framework

Dynamic hybrid RANS-LES (DHRL) is used to implement the WMRLES into general-purpose computational fluid dynamics solvers. The incompressible form of the momentum equation for the DHRL formulation used here can be expressed as:

(7) $$\frac{\partial u_{i}}{\partial t}+\frac{\partial \left(u_{i}u_{j}\right)}{\partial x_{j}}=-\frac{1}{\rho }\frac{\partial P}{\partial x_{i}}+\frac{\partial }{\partial x_{j}}\left(2\nu S_{ij}\right)+\frac{\partial }{\partial x_{j}}\left(\sigma_{ij,SGS}\right)+\frac{\partial }{\partial x_{j}}\left[\left(1-\alpha \right)\left(\sigma_{ij,RANS}-{\bar{\sigma }}_{ij,SGS}\right)\right],$$

where ρ, $u_{i}$, and P are the resolved (implicitly filtered) density, velocity, and pressure, μ is the dynamic viscosity, and $S_{ij}$ is the strain-rate tensor:

(8) $$S_{ij}=\frac{1}{2}\left(\frac{\partial u_{i}}{\partial x_{j}}+\frac{\partial u_{j}}{\partial x_{i}}\right).$$

The straight overbar denotes a Reynolds-averaged quantity. The modeled stress tensor $\sigma_{ij,SGS}$ is obtained from an appropriate subgrid stress model and is zero for monotonicity-preserving implicit LES (MILES). In general, the modeled stress tensor $\sigma_{ij,RANS}$ is obtained from an appropriate Reynolds-averaged Navier–Stokes turbulence model, for which the model equations are solved using the Reynolds-averaged variable field.

The variable α is a spatially varying blending parameter that governs the RANS-to-LES transition of the turbulent stress term in the momentum equation. In regions of the domain where α=1, the unmodified form of the LES model equations is utilized; in regions with α=0, a mean flow solution is obtained using the RANS model. Regions for which 0<α<1 represent the transitional regions between the two.

In the original formulation of DHRL, the blending function served to ensure that the total (modeled plus resolved) turbulent production in the transition region was equal to or greater than the production that would be predicted by the RANS model alone. The blending parameter is computed locally as:

(9) $$\alpha =\frac{\underset{\begin{array}{c} Resolved\;turbulent\\ production \end{array}}{-\underbrace{\bar{u_{i}^{\prime \prime }u_{j}^{\prime \prime }}\;}{\bar{S}}_{ij}}}{\underset{\begin{array}{c} RANS\;\\ Production \end{array}\;}{\underbrace{\sigma_{ij,RANS}{\bar{S}}_{ij}}}-\underset{\begin{array}{c} Inhomogeneous\\ SGS\;Production \end{array}}{\underbrace{{\bar{\sigma }}_{ij,SGS}\;{\bar{S}}_{ij}}}},$$

and, in practice, is limited to a minimum value of zero and a maximum value of one. The DHRL framework was formulated to address the effects of modeled stress depletion in the transition regions for which the level of resolved fluctuating velocity was not sufficient to produce a well-resolved LES result. It is apparent from Equation (9) that when the resolved fluctuating velocity in a simulation is locally zero, then the value of α limits to zero. For traditional hybrid RANS-LES simulation, this commonly includes freestream regions or attached boundary layers upstream of the separation, where the solution is steady-state in a numerical sense. Observing Equation (7), in that case, a pure RANS simulation is recovered in that region of the computational domain, since the momentum equation reduces to:

(10) $$\frac{\partial u_{i}}{\partial t}+\frac{\partial \left(u_{i}u_{j}\right)}{\partial x_{j}}=-\frac{1}{\rho }\frac{\partial P}{\partial x_{i}}+\frac{\partial }{\partial x_{j}}\left(2\nu S_{ij}\right)+\frac{\partial }{\partial x_{j}}\left(\sigma_{ij,RANS}\right).$$

In contrast, when the fluctuating velocity field produces resolved turbulent production that is sufficiently high, the numerator on the right-hand-side of Equation (9) becomes equal to or greater than the denominator, and the value of α is equal to or limited to one. Observing Equation (7) once again, that condition yields a pure LES simulation in those regions of the domain, as the momentum equation becomes:

(11) $$\frac{\partial u_{i}}{\partial t}+\frac{\partial \left(u_{i}u_{j}\right)}{\partial x_{j}}=-\frac{1}{\rho }\frac{\partial P}{\partial x_{i}}+\frac{\partial }{\partial x_{j}}\left(2\nu S_{ij}\right)+\frac{\partial }{\partial x_{j}}\left(\sigma_{ij,SGS}\right),$$

In regions where the velocity fluctuations are non-zero but not highly resolved, the value of α lies between zero and one, and the simulation operates in a transition zone between LES and RANS. One primary drawback of the original DHRL formulation is that the value of α can be less than one in any region of the flowfield, so the inclusion of the RANS model component is not restricted to the near-wall region only, and the RANS contribution may indeed be significant even in separated or free shear flow regions. For the wall treatment approach proposed here, that drawback is mitigated through the use of a grid-based limit on the RANS eddy viscosity, as discussed in the following section.

#### 2.1.2. Mixing Length Eddy Viscosity Model

The DHRL-based WMRLES strategy proposed here is implemented using an algebraic mixing length model to obtain the RANS component in the DHRL framework, where the mixing length is limited to ensure that the RANS contribution diminishes to zero far from the wall. A model similar to the Prandtl [27] mixing length model with van Driest [28] type damping is adopted. The general model form is:

(12) $$\sigma_{ij,RANS}=2\nu_{T,RANS}{\bar{S}}_{ij},$$

(13) $$\nu_{T,RANS}=l_{m}^{2}\bar{S},$$

(14) $$l_{m}=f_{\mu }\kappa d^{*},$$

(15) $$f_{\mu }=1-exp\left[-{\left(\frac{{Re}_{y}}{A_{\mu }}\right)}^{2}\right],$$

(16) $${Re}_{y}=\frac{d^{2}\bar{S}}{\nu },$$

(17) $$\bar{S}=\sqrt{2{\bar{S}}_{ij}{\bar{S}}_{ij}}.$$

Note that the damping function is based on the local variable ${Re}_{y}$ rather than $y^{+}$. The value of the von Karman constant κ for the simulations reported here was 0.384 based on the results of Lee and Moser [29]. This value was selected for the sake of consistency with the primary test case used in this study. The model coefficient $A_{\mu }$ was then set to 70 based on the results from a RANS implementation of the mixing length model which was used for calibration purposes. An alternative formulation was obtained through a similar calibration process, for which κ=0.4 and $A_{\mu }=75$. Though not shown, the WMRLES results using the alternative formulation were nearly indistinguishable from those shown in the Results section below using κ=0.384.

In the standard mixing length model, the relevant length scale is the wall distance, d, which is defined at each location in the mesh as the shortest distance from that point to any wall boundary. For the WMRLES approach proposed here, the effect of the RANS contribution in the near-wall region should vanish in the outer layer and in the separated shear layer or freestream regions to ensure that the model operates as a pure LES. To accomplish this, the length scale is limited based on the characteristic local mesh spacing, ∆:

(18) $$d^{*}=min\left(d,C_{d}∆\right).$$

The local mesh size for the finite-volume simulations presented here is defined for a given computational control volume (cell) as the maximum distance between the cell centroid and all neighbor cell centroids. Limiting the effective wall distance based on grid size ensures that far from the wall, the mixing length is limited, and the eddy viscosity (Equation (13)) is reduced relative to the standard mixing length model. The form of Equation (18) ensures that the concept of “far from the wall” is expressed relative to the local mesh size, and the value of the coefficient $C_{d}$ determines precisely where the limit on wall distance is imposed. For example, if the value of $C_{d}$ is 4, then the mixing length will be limited to be no greater than the size of four mesh cells. In practice, the appropriate value of $C_{d}$ will depend on the mesh topology and numerical scheme. The effect of this coefficient is investigated in the present study, but it is expected that it should be approximately 2–3 based on the so-called third grid-point method for the matching of the wall function in traditional WMLES methods [30].

### 2.2. Mesh Resolution Estimates

Following the analysis of Choi and Moin [2], it is possible to derive general mesh resolution estimates for WMRLES in which the near-wall mesh is resolved to arbitrary level, i.e., the first-cell $y^{+}$ may be specified to any value depending on the level of resolution required for computation of the mean velocity profile in the near field. Resolution levels in the streamwise and spanwise directions are assumed to be identical to traditional WMLES, while grid stretching is employed in the wall-normal direction. For a boundary layer height δ, the characteristic mesh size for WMLES is defined as $∆x=\delta /n_{x}$, $∆y=\delta /n_{y}$, and $∆z=\delta /n_{z}$, where x, y, and z denote the streamwise, wall-normal and spanwise coordinate directions, respectively, and $n_{i}$ is the number of mesh cells used to resolve a length δ in a particular direction.

For the present analysis, we define an inner mesh layer of size $\delta_{I}$, and a corresponding outer layer of size $\delta_{O}=\delta -\delta_{I}$. Uniform spacing ∆x and ∆z is assumed throughout the boundary layer, uniform spacing ∆y is assumed in the outer layer, and variable wall-normal spacing is applied in the inner layer.

Mesh resolution in the inner layer is characterized by $y_{w}^{+}$ and the wall-normal stretching ratio r, which is defined such that the cell size distribution in the inner layer is:

(19) $$∆y_{m+1}=r∆y_{m},$$

where the increasing value of the index m indicates increasing integer cell distance from the wall. The number of wall-normal cells $n_{I}$ in the inner region can then be computed based on the ratio of the viscous length scale $\nu /u_{\tau }$ and the boundary layer height δ as:

(20) $$n_{I}=\frac{\ln\left[\left(\frac{∆y}{\delta }\right)\left(\frac{{Re}_{\tau }}{2y_{w}^{+}}\right)\right]}{\ln r},$$

where ${Re}_{\tau }=\frac{u_{\tau }\delta }{\nu }$. Note that in the context of finite-volume simulations, $y_{w}^{+}$ denotes the wall distance of the first-cell centroid, so the overall first-cell height is $2y_{w}^{+}$.

For meshes constructed in this manner, the total number of boundary layer cells $N_{y}$ in the wall-normal direction can be obtained as:

(21) $$N_{y}=n_{I}+n_{O},$$

(22) $$n_{O}=\frac{\delta_{O}}{∆y}=\frac{\delta -\delta_{I}}{\delta }n_{y}.$$

The inner layer height is computed as:

(23) $$\frac{\delta_{I}}{\delta }=\left(\frac{r^{n_{I}}-1}{r-1}\right)\left(\frac{2y_{w}^{+}}{{Re}_{\tau }}\right),$$

and the number of cells in the outer layer is:

(24) $$n_{O}=n_{y}\left[1-\left(\frac{r^{n_{I}}-1}{r-1}\right)\left(\frac{2y_{w}^{+}}{{Re}_{\tau }}\right)\right].$$

Since the streamwise and spanwise spacings between WMRLES and WMLES are identical, the ratio of total boundary layer cell count between the two methods can be simply expressed as:

(25) $$R=N_{y}/n_{y}.$$

For example, given a boundary layer with ${Re}_{\tau }=4000$, a desired near-wall resolution $y_{w}^{+}=1$, inner layer stretching ratio r=1.2, and outer layer resolution of $n_{y}=20$, then the mesh increase factor for WMRLES versus WMLES with uniform wall-normal cell distribution can be found by:

(26) $$n_{I}=\ln\left[\left(\frac{1}{20}\right)\left(\frac{4000}{2}\right)\right]/\ln\left(1.2\right)=25.25\approx 25,$$

(27) $$n_{O}=20\left[1-\left(\frac{1.2^{25.25}-1}{1.2-1}\right)\left(\frac{2}{4000}\right)\right]=15.05\approx 15,$$

(28) $$R=\frac{n_{I}+n_{O}}{n_{y}}=2.$$

Using the methodology in [2], the overall mesh requirements for a developing boundary layer can be obtained by employing the estimates:

(29) $$\frac{\delta }{x}=0.16{Re}_{x}^{-1/7},$$

(30) $$c_{f}=0.027{Re}_{x}^{-1/7},$$

and integrating over the boundary layer region:

(31) $$N_{LES}=\int_{0}^{L_{x}}\int_{0}^{L_{z}}\frac{n_{x}N_{y}n_{z}}{\delta^{2}}\;dx\;dz.$$

Since the wall-normal mesh resolution requirement (in terms of cell count) increases with Reynolds number, a conservative (upper bound) on the cell count ratio R can be found using the above expression for $N_{y}$ and assuming a value of the length-scale ratio ${Re}_{\tau }$ based on the maximum Reynolds number ${Re}_{L}$. Based on the above empirical relations:

(32) $${Re}_{\tau ,max}=0.0186\;{Re}_{L}^{11/14}.$$

It is therefore possible for any given Reynolds number to determine the relative increase in mesh count required to use WMRLES versus WMLES. Table 1 shows the estimated mesh requirement for $y_{w}^{+}=1$ and two different values of stretching ratio r. The example case is the same as in Ref. [2] of flow over an airfoil without separation. The assumed outer resolution is equal to that which was first recommended by Chapman [31], i.e., $n_{x}=n_{z}=10$, $n_{y}=25$.

As shown in Table 1, for a mesh with near-wall resolution $y_{w}^{+}=1$, the increase in cell count for WMRLES versus that of WMLES with uniform wall-normal spacing, in terms of the ratio R, ranges from 1.28 to 2.63. In contrast, WRLES with an equivalent wall-normal mesh spacing increases the cell count versus WMLES from R=1.44 at ${Re}_{C}=10^{6}$ to R=469 at ${Re}_{C}=10^{9}$. WMRLES with variable wall-normal spacing may therefore, in theory, be realized with only an approximate doubling of the overall mesh count, while potentially eliminating the need for specialized wall function boundary conditions and allowing some level of resolution of near-wall turbulent velocities that impact the fluctuating temperature and heat flux behavior at the fluid–wall interface.

### 2.3. Simulation Details

Validation simulations were performed using Loci-CHEM [32,33], an open-source, density-based, three-dimensional finite-volume flow solver on general polyhedral meshes. Discretization of the convective flux terms was performed using a blended scheme with second-order MUSCL extrapolation of the left and right face states from the neighboring cells using least squares gradients with slope limiting for stability [34]. The flux, F, is constructed as the sum of a centered and an upwind portion:

(33) $$F=\eta \;F_{upwind}+\left(1-\eta \right)\;F_{centered},$$

where the upwind weighting parameter η varies between zero and one. The centered contribution is obtained using a kinetic energy consistent formulation [35] and the upwind contribution is a locally Mach-number weighted combination that limits to the simple upwinding of the convective terms [34] for Ma=0 and limits to the HLLC (Harten–Lax–van Leer contact) flux [36] for Ma≥1. The scheme is formally second-order accurate but has been found to yield low numerical dissipation and to be well suited for scale-resolving simulations of turbulent flow. The effect of varying the weighting parameter η between the centered and upwind component of the flux is investigated in the current study to determine the effect on the proposed wall-modeling method.

To compute the time-dependent solution, unsteady terms in the governing equations were discretized using a second-order three-point backward difference formulation, and the simulation was advanced using implicit time marching. Numerical experiments were performed to determine the appropriate time step size, ∆t. It was found that results were fully independent of ∆t for:

(34) $$∆t\leq 0.5\frac{∆x}{U_{cl}},$$

where ∆x is the characteristic mesh spacing in the streamwise direction, and $U_{cl}$ is the channel centerline mean velocity.

Since the channel flow test case in the present study is a stationary turbulent flow, running time-averaging was used to compute the Reynolds-averaged quantities in the DHRL framework. For an arbitrary variable φ, the time-averaged value $\bar{\varphi }$ at a given position and time step n is computed as:

(35) $${\bar{\varphi }}_{n}=\frac{1}{n}\left[\left(n-1\right){\bar{\varphi }}_{n-1}+\varphi_{n}\right].$$

During the simulation, Reynolds-averaged quantities are obtained solely from the running time average defined in Equation (35).

### 2.4. Channel Flow Test Cases

The initial demonstration test case for the proposed WMRLES method is turbulent plane channel flow at ${Re}_{\tau }=5200$ and 2000, and matches the DNS simulations performed by [29,37]. The computational domain had the channel half-height δ and overall extent, where $L_{x}\times L_{y}\times L_{z}=4\pi \delta \times 2\delta \times 1.5\pi \delta$. Two different base Cartesian structured meshes were used, namely a coarse mesh with boundary layer nominal cell count $n_{x}=8$, $n_{y}=n_{z}=16$, as well as a fine mesh with $n_{x}=16$, $n_{y}=n_{z}=24$. These were selected based on the range of mesh sizes used for WMLES in the previous studies reported in [2], who had suggested $n_{x}=5-32$, $n_{y}=16-32$, and $n_{z}=15-32$. For the coarse mesh the wall-normal size of the first cell off the wall was selected such that the cell centroid was located at either $y_{w}^{+}=1$ or $y_{w}^{+}=2$, to investigate the effect of first-cell height on the results. In the near-wall region, the default wall-normal stretching ratio used was r=1.2. On the coarse base mesh, two additional near-wall meshes were constructed using r=1.33 and r=1.5 to investigate the effect of wall-normal stretching on the results.

All grids were constructed using the guidelines outlined in Section 2.2. For example, for the ${Re}_{\tau }=5200$ case, with a coarse base mesh, $y_{w}^{+}=1$, and r=1.2, the outer region wall-normal mesh size was ∆y=δ/16, and the number of inner layer cells required was found from Equation (20) to be $n_{I}=28$. The inner layer extent was found from Equation (23) to be $\delta_{I}=0.315$ with a corresponding outer layer size of $\delta_{O}=0.685$, which were adjusted to $\delta_{I}=0.3125$ and $\delta_{O}=0.6875$ to accommodate an integer number of outer layer cells of size ∆y=δ/16. The number of cells in the outer layer was 11 and the total number in the wall-normal direction for the channel half-height was 39. The number of cells required versus traditional WMLES with wall functions was therefore increased by a ratio of 39/16 = 2.44. For the corresponding fine mesh with $y_{w}^{+}=1$, and r=1.2, ∆y=δ/24, $n_{I}=26$, $\delta_{I}=0.2083$, and $n_{O}=19$, with a ratio cell count increase over traditional WMLES of 45/24 = 1.88. The increase in mesh size for WMRLES versus WMLES is shown for all simulations in Table 2. Grids A through H in Table 2 are for the channel test case with ${Re}_{\tau }=5200$, while grid I is for the channel test case with ${Re}_{\tau }=2000$.

**Figure 1:**
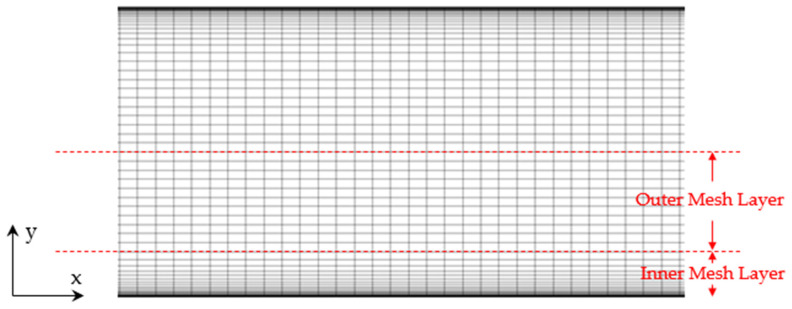
Illustration of near-wall meshing strategy for channel flow test case.

Figure 1 shows one of the coarse WMRLES meshes (Grid B: $n_{y}=16$, $y_{w}^{+}=1$, r=1.2) in order to illustrate the meshing strategy outlined in Section 2.2. The outer layer has uniform mesh in all three coordinate directions, while the inner layer is stretched in the wall-normal direction to resolve the mean velocity close to the wall. 

## 3. Results

Several different simulations of the turbulent channel flow test case were performed, as shown in Table 3. Results were first obtained using the coarse grid in order to perform a systematic investigation into the effect of mesh, numerical scheme, subgrid stress model, and mesh-based limiting value used in the mixing length model ($C_{d}$ in Equation (16)). The effect of the numerical scheme was evaluated by varying the degree of upwinding used in the hybrid upwind–centered convective flux reconstruction. The effect of the subgrid stress model was evaluated by comparing the results obtained with the monotonicity-preserving implicit large-eddy simulation (MILES) [38] and the wall-adapting local eddy viscosity (WALE) explicit model [39]. Based on observations from the initial coarse grid simulations, an appropriate set of operating parameters was selected, and results were obtained on the fine grid to illustrate the effect of the base grid resolution. Simulations were also performed using wall function-based WMLES, as well as with three different hybrid RANS-LES models, to compare against the WMRLES results.

**Figure 2:**
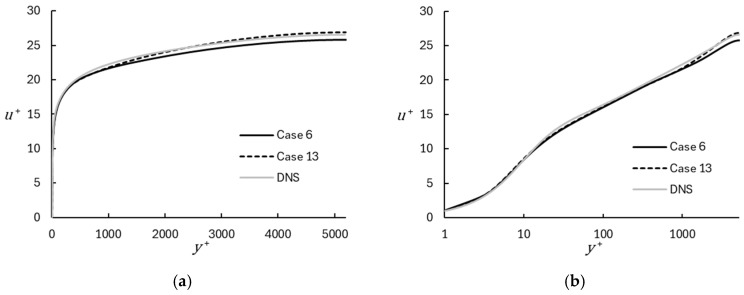
Mean velocity profiles for representative coarse and fine mesh cases using WMRLES, with comparison to DNS results [29]: (**a**) Linear; (**b**) Semilog.

**Figure 3:**
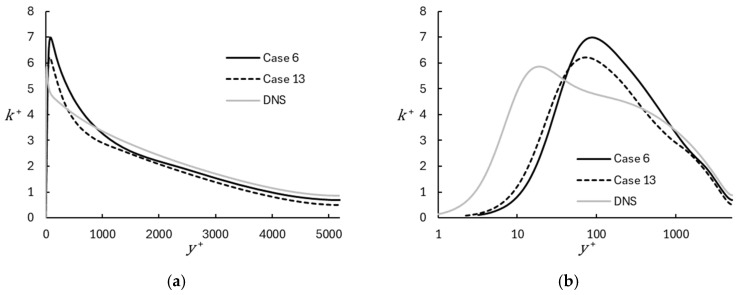
Turbulent kinetic energy profiles for representative coarse and fine mesh cases using WMRLES, with comparison to DNS results [29]: (**a**) Linear; (**b**) Semilog.

**Figure 4:**
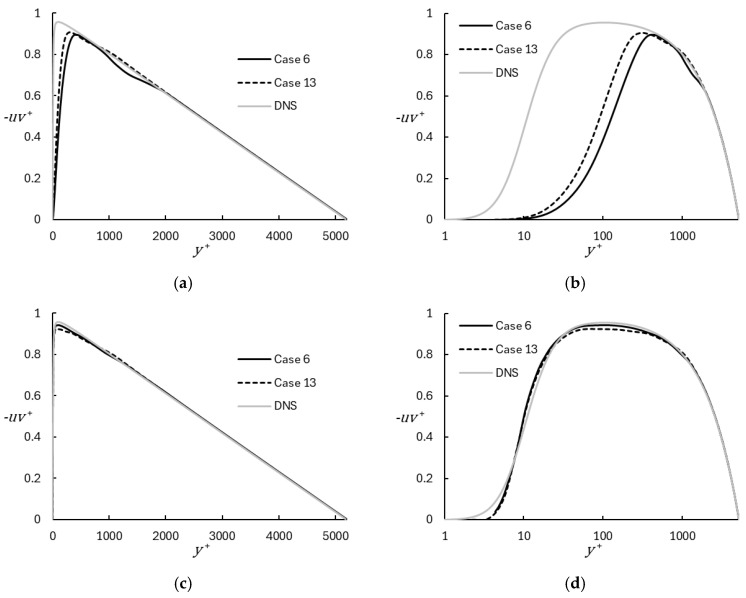
Reynolds shear stress profiles for representative coarse and fine mesh cases using WMRLES, with comparison to DNS results [29]: (**a**) Resolved stress (Linear); (**b**) Resolved stress (Semilog); (**c**) Sum of resolved and modeled stress (Linear); (**d**) Sum of resolved and modeled stress (Semilog).

**Figure 5:**
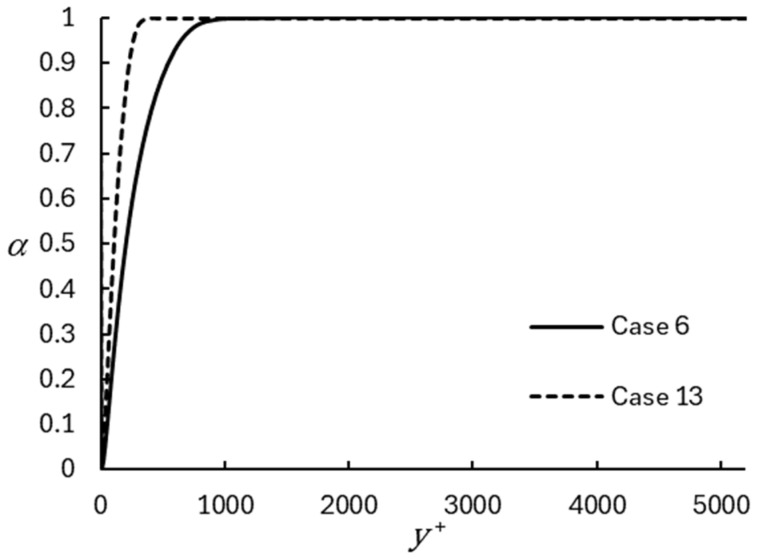
Distribution of the DHRL blending function, α. The mixing length RANS model is only active in a region close to the wall.

**Figure 6:**
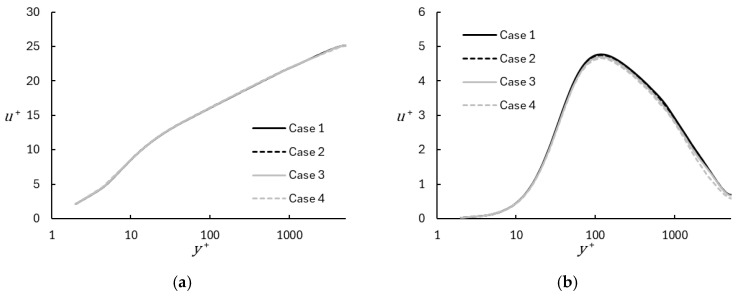
Mean velocity (**a**) and turbulent kinetic energy (**b**) profiles for cases with different values of the mixing length limiting coefficient, $C_{d}$.

**Figure 7:**
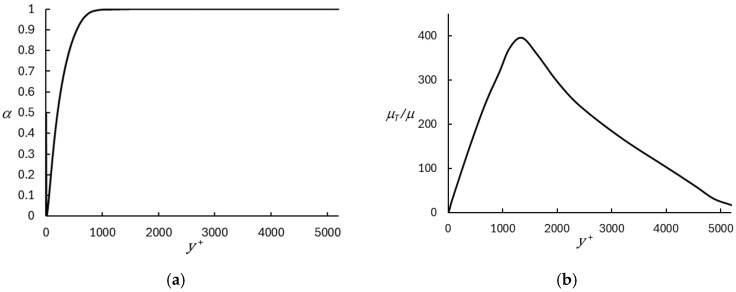
Profiles of DHRL blending parameter (**a**) and normalized turbulent viscosity (**b**) for Case 2. The grid-based RANS mixing length limit is applied sufficiently far from the wall that the simulation is fully LES at that location.

**Figure 8:**
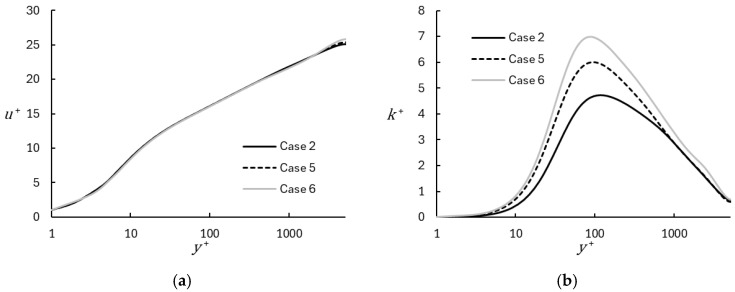
Profiles of mean velocity (**a**) and turbulent kinetic energy (**b**) comparing results obtained with different upwind weighting in the convective flux term. Case 1 is most numerically dissipative; Case 6 is least numerically dissipative.

**Figure 9:**
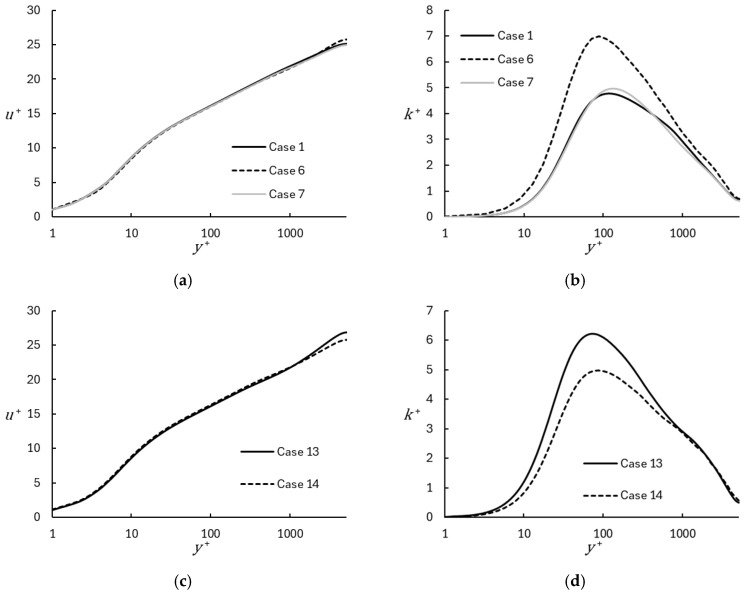
Illustration of the effect of subgrid model. Cases 7 and 14 were run using the WALE explicit model, Cases 1, 6, and 13 were run using MILES: (**a**) Mean velocity, coarse grid; (**b**) Turbulent kinetic energy, coarse grid; (**c**) Mean velocity, fine grid; (**d**) Turbulent kinetic energy, fine grid.

**Figure 10:**
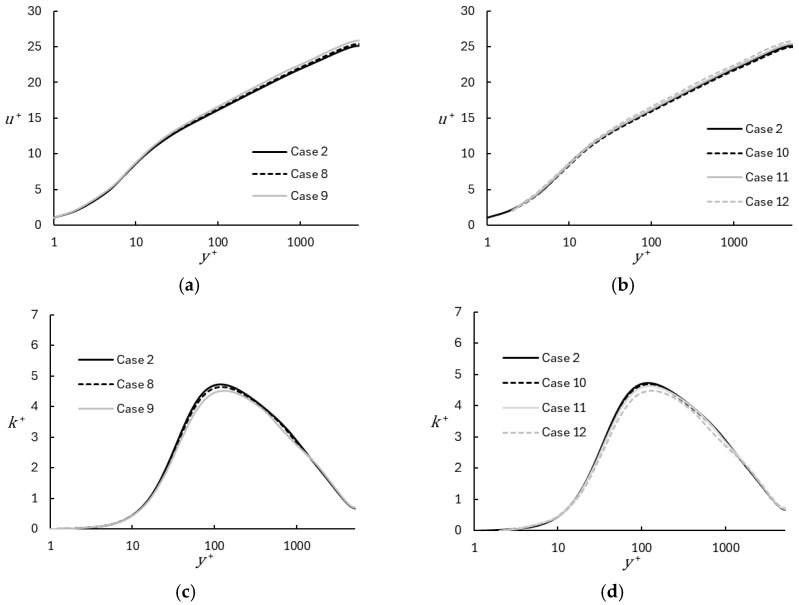
Profiles of mean velocity and turbulent kinetic energy using the coarse base grid and different near-wall meshing parameters: (**a**,**c**) Effect of varying wall-normal stretching ratio with $y_{w}^{+}=1$; (**b**,**d**) Effect of varying wall-normal stretching ratio with $y_{w}^{+}=2$.

**Figure 11:**
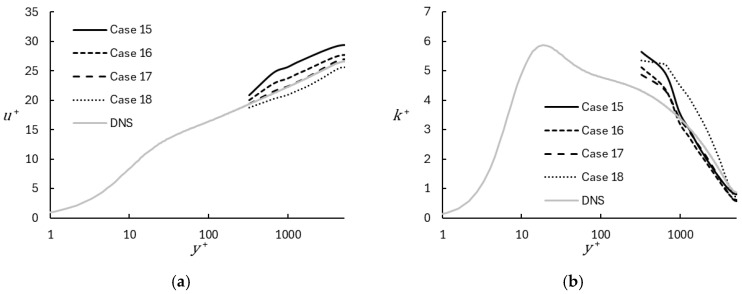
Profiles of mean velocity (**a**) and turbulent kinetic energy (**b**) comparing results obtained using traditional WMLES. Different cases represent different levels of upwinding in the convective flux term or different subgrid stress models.

**Figure 12:**
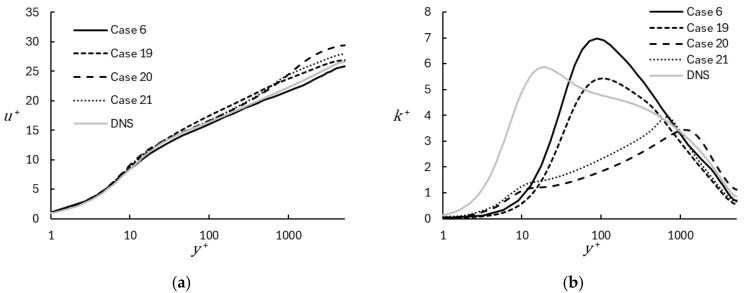
Profiles of mean velocity (**a**) and turbulent kinetic energy (**b**) comparing results obtained using DHRL, DES, IDDES, and WMRLES.

**Figure 13:**
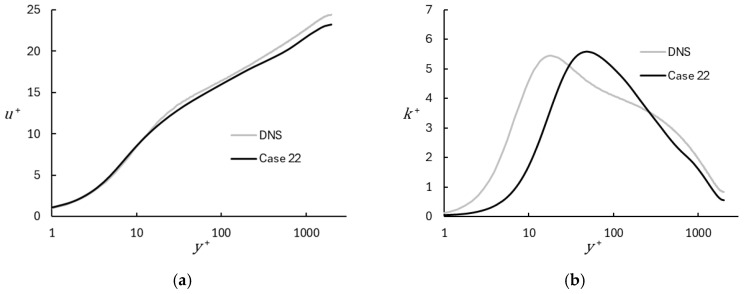
Comparison of WMRLES results with DNS data [37] for the case of ${Re}_{\tau }=2000$: (**a**) mean velocity; (**b**) turbulent kinetic energy.

The results are presented in Figure 2, Figure 3, Figure 4, Figure 5, Figure 6, Figure 7, Figure 8, Figure 9, Figure 10, Figure 11, Figure 12 and Figure 13. Comparisons are primarily made in terms of mean velocity and turbulent kinetic energy for each test case. Statistical mean and variance were computed using the running time average (Equation (35)), and an additional planar averaging step was applied during post-processing. Figure 2 shows a representative comparison of mean velocity between the WMRLES results on the coarse and fine base grids with the DNS data. The results follow the expected trend in the viscous sublayer, logarithmic region, and wake region, although the deviation of the wake from the log-law is less pronounced for WMRLES than for DNS. Importantly, there is no evidence of log-layer mismatch, which indicates that the DHRL blending approach allows for a smooth transition between the RANS-dominated near-wall region and full LES outer region. Not surprisingly, results obtained on the finer grid (Case 13) show better agreement with DNS than results on the coarse grid (Case 6).

Profiles of turbulent kinetic energy for the representative coarse and fine grid WMRLES cases are compared to the DNS results in Figure 3. The turbulent kinetic energy is shown using inner scaling:

(36) $$k^{+}=\frac{1}{2u_{\tau }^{2}}\left(\bar{{u^{\prime }}^{2}}+\bar{{v^{\prime }}^{2}}+\bar{{w^{\prime }}^{2}}\right).$$

The general trend is reproduced on both grids, including a peak near the wall and monotonic decrease towards the channel centerline. The peak distance from the wall and its value are overpredicted, since the peak in the DNS occurs in the buffer layer, which is under-resolved in the WMRLES simulations. Consequently, the WMRLES simulations show an artificial buffer layer at a wall distance proportional to the mesh size. Note that the peak moves closer to the wall for the fine mesh since the mesh in this case allows for the resolution of smaller turbulent scales. If mesh refinement were to continue, the peak would be expected to continue to approach the correct location until the DNS refinement limit is reached. The overprediction of the peak value in the WMRLES results is also consistent with the model implementation, since the added dissipation from the RANS contribution in the near-wall region is applied only to the mean flow, as seen in Equation (7). Near-wall turbulent dissipation is primarily due to numerical dissipation for the MILES simulations, or to the SGS model in the WALE simulations. The results shown below confirm that increasing either the numerical or SGS dissipation yields a lower peak value of turbulent kinetic energy in the near-wall region. Notably, the differences between the DNS and WMRLES predictions of resolved turbulent kinetic energy appear to have a relatively small effect on the mean velocity profiles as shown in Figure 2. This robustness of the mean flow results to the details of the numerical scheme and/or SGS model is a feature of the DHRL implementation that has been previously demonstrated [8,9,10,11].

For the channel flow test case turbulent transport of mean momentum is due exclusively to the $\bar{u^{\prime }v^{\prime }}$ component of the Reynolds shear stress. Comparison of the shear stress profile with DNS results is presented in Figure 4, where:

(37) $${uv}^{+}=\frac{\bar{u^{\prime }v^{\prime }}}{u_{\tau }^{2}},$$

Figure 4a,b show the resolved shear stress from the LES portion of the simulation. It is apparent that the mesh is insufficiently resolved to correctly reproduce the shear stress near the wall, and that the region in which this occurs is reduced for the finer mesh. Figure 4c,d highlight the effect of the near-wall model contribution, showing the total shear stress including both the resolved and modeled portion, where the modeled portion is due to the eddy viscosity introduced by the mixing length model. For both the coarse and fine meshes, the results are significantly improved and show consistent agreement with the DNS results.

The blending between RANS and LES, and therefore the extent of the wall modeled region, is shown in Figure 5 for the same two representative cases. The plots present the distribution of the DHRL blending parameter, α, which smoothly varies from a value near zero (indicating that the model is acting in RANS mode) at the wall to a value of one (indicating that the model is acting in LES mode) at some fraction of the channel half-height. For both the coarse and fine grid cases, a majority of the channel is resolved using a pure LES, with no influence of the mixing length RANS model. The model operates as a pure LES for approximately $\frac{y}{\delta }>0.2$ for the coarse mesh, as well as for approximately $\frac{y}{\delta }>0.08$ for the fine mesh. This is because the fine mesh is able to accurately resolve the large-eddies responsible for mean momentum transfer and turbulent production closer to the wall than the coarse mesh. Similar distributions of α were obtained for all the simulations performed.

As described in Section 2.1.2, the mixing length used in the algebraic eddy viscosity model is limited based on the local mesh size, in order to constrain its influence to the near-wall region. The value of the limit is determined by the model coefficient, $C_{d}$ (Equation (18)). The influence of this coefficient is shown in Figure 6, which presents the mean velocity and turbulent kinetic energy profiles for different values of $C_{d}$ ranging from one to four. It is apparent that the results are relatively insensitive to $C_{d}$, with only a small difference discernable in the case of $C_{d}=4$. In practice, it is desired to minimize the influence of the RANS model while still ensuring that it is not limited to too close to the wall. It was determined that $C_{d}=2$ represented a reasonable compromise. This is illustrated in Figure 7, which shows the profiles of the DHRL blending function (Figure 7a) and profiles of the eddy viscosity in the mixing length model (Figure 7b). The peak in eddy viscosity at approximately $y^{+}=1300$ indicates the location at which the mixing length is limited by the local mesh size. Comparison with the profile of α shows that this occurs outside of the RANS influenced near-wall layer, in the region where α=1, as desired. The figure also shows that the region of the domain in which any contribution of the RANS model is present is limited to the near-wall region, as desired.

The effect of numerical dissipation due to the upwind portion of the convective flux is shown in Figure 8 for the upwind values of η = 0.05, 0.1, and 0.2. The effect on the mean velocity shown in Figure 8a is minimal, with only small differences apparent in the wake region. As expected, the effect on resolved turbulent fluctuations is greater, since increased dissipation results in an effectively larger implicit filtering of the turbulent spectrum, as well as the decreased resolution of eddy structures with scales comparable to the local grid size [38]. This is apparent in Figure 8b, where the peak resolved turbulent kinetic energy increases and moves closer to the wall as the degree of upwinding is decreased. Importantly, as the resolved turbulent kinetic energy and, consequently, turbulent stress are increased, the dynamic blending function (Equation (9)) responds in an appropriate manner to produce a consistent prediction of the mean velocity distribution in the near-wall region.

Two simulations were run with the WALE explicit subgrid stress model and compared to simulations run with MILES. For the WALE cases, the upwind portion of the connective flux was set to 0.05, consistent with Case 6. The WALE results were obtained on both coarse and fine meshes and are shown in Figure 9. It is apparent that the use of the WALE model yields a level of dissipation in the resolved scales that is comparable to approximately η = 0.2 upwinding in the MILES simulations. The MILES simulations with lower upwinding yield significantly higher levels of resolved turbulence as indicated in Figure 9b,d. The effect on the mean velocity is apparent in Figure 9a,c. The plots show that the effect of the WALE SGS model is to reduce the wake region, with negligible effect in the log-layer region. Overall, the inclusion of the WALE model has a similar effect on the simulation as using MILES with a higher level of numerical dissipation. This is not surprising since the WALE model simply adds a subgrid eddy viscosity and therefore manifests as additional diffusion in the simulations.

Finally, the effect of the mesh characteristics in the near-wall region is shown in Figure 10. As described in Table 2, the wall-normal mesh in the near-wall region was constructed with three different values of stretching ratio and two different values for first cell $y_{w}^{+}$. In Figure 10a, it is seen that the use of a larger stretching ratio yields a slight increase in the predicted mean velocity in the log-layer and wake regions, although the increase is relatively small even for a change in stretching ratio from 1.2 to 1.5. Similarly, increasing the first cell height by a factor of two from $y_{w}^{+}=1$ to $y_{w}^{+}=2$ results in almost no change in the mean velocity profile (Case 2 vs. Case 10). It is certain that a continued increase in first cell height would lead to a noticeably detrimental effect on the mean velocity result, but the simulations appear to show little sensitivity in the range investigated. The effect of mesh details on the turbulent kinetic energy was also relatively small as seen in Figure 10c,d.

As listed in Table 3, comparison simulations were performed using a traditional WMLES approach, and the results are shown in Figure 11. For these cases, the built-in WMLES model in Loci-CHEM was used, which applied a wall shear stress boundary condition based on the instantaneous velocity in the first cell from the wall, according to the universal near-wall velocity profile of Spalding [16]:

(38) $$y^{+}=u^{+}+e^{-\kappa B}\left[e^{\kappa u^{+}}-1-\kappa u^{+}-\frac{1}{2}{\left(\kappa u^{+}\right)}^{2}-\frac{1}{6}{\left(\kappa u^{+}\right)}^{3}-\frac{1}{24}{\left(\kappa u^{+}\right)}^{4}\right],$$

During each time step and at each wall boundary face, Equation (38) was iterated until a converged value of the friction velocity was obtained, which was then used to specify the wall boundary condition. Cases 15–17 were run using MILES as the SGS model, with different percentages for the contribution of the upwind portion of the convective flux term. Case 17, which uses upwinding η = 0.05, showed very good agreement for mean velocity with the DNS results, while Cases 15 and 16 showed the well-known log-layer mismatch. Case 18 used upwinding η = 0.05 and the WALE SGS model and, interestingly, showed an underprediction of the mean velocity compared to DNS. With regard to the turbulent kinetic energy, the three cases using MILES showed similar results and reasonable agreement with DNS, comparable to the WMRLES results for $y^{+}>1000$, while the case which used the WALE model overpredicted the fluctuating energy. Overall, the results highlight the sensitivity of WMLES to the numerical and modeling details of the computational formulation.

The performance of the WMRLES model is compared to several other hybrid RANS-LES models, namely DES, IDDES, and standard DHRL with the k-ω SST RANS model. These comparisons can be seen in Figure 12, where results are shown for the mean velocity profile and the turbulent kinetic energy profile for all model types. As seen in Figure 12a, the WMRLES most closely matches the DNS results with a minor underprediction of the velocity profile. When comparing the kinetic energy profiles in Figure 12b, the WMRLES model predicts a higher peak value off the wall. All the other models underpredict the kinetic energy profile and overpredict the mean velocity profile. The DES and IDDES models both overpredict the velocity in the center of the channel, though IDDES shows improvement over DDES as expected. The WMRLES model is able to very accurately predict the viscous sublayer and the log-layer region, with a tendency to slightly underpredict the mean velocity throughout the domain. The results indicate that the method proposed here yields results comparable to traditional hybrid RANS-LES models, with a reduced computational cost due to the use of a zero-equation RANS model component.

To investigate the generalizability of the WMRLES model, a second channel flow case was investigated with a different Reynolds number, matching the DNS results of Hoyas and Jimenez [37]. The simulation was run using model and numerical settings equivalent to Case 6. Figure 13 shows the mean velocity and turbulent kinetic energy profiles obtained with WMRLES. The WMRLES model slightly underpredicts the mean velocity profile in the middle region of the channel, similar to the cases with ${Re}_{\tau }=5200$. Additionally, the peak value of turbulent kinetic energy is slightly overpredicted and is predicted further off the wall than in the DNS, and the near-wall turbulent kinetic energy values are underpredicted, all of which are similar to the higher Reynolds number case.

## 4. Conclusions

A simple methodology for near-wall modeling in large-eddy simulations was presented, denoted in this paper as wall mean-resolved large-eddy simulation (WMRLES). The method is based on the blending of the RANS and LES simulation modes using the dynamic hybrid RANS-LES (DHRL) framework, adopting an algebraic mixing length eddy viscosity model for the RANS component, and limiting the model mixing length based on the local mesh size. A near-wall mesh strategy to minimize the increase in mesh count compared to traditional wall-modeled large-eddy simulation (WMLES) was also proposed. A comprehensive set of initial validation results were presented for turbulent channel flow at ${Re}_{\tau }=5200$, with an additional validation test case performed for ${Re}_{\tau }=2000$. Key conclusions are outlined in the following points:

- The proposed near-wall mesh strategy allowed for the implementation of WMRLES using approximately two times the required mesh resolution for WMLES. For the channel flow cases, the relative increase for WMRLES ranged from 1.56 to 2.44.
- Results using the WMRLES model, in terms of mean velocity and turbulent kinetic energy, were compared to DNS data and found to be comparable to similar results obtained with other near-wall methods such as WMLES, DHRL, DES, or IDDES. The results allowed for the full resolution of the mean flow to the wall, requiring neither algebraic nor numerical wall functions, and did not show any evidence of log-layer mismatch.
- The WMRLES method was found to be robust and relatively insensitive to model limiting coefficient, numerical dissipation, subgrid stress model, and near-wall mesh resolution characteristics. The most significant effect was seen to be due to increased numerical dissipation due to increased upwinding in the convective flux terms.
- The method can be implemented in a straightforward manner into existing general-purpose turbulent flow solvers.

## Figures and Tables

**Table 1:** Estimated mesh cell count for boundary layer flow over an airfoil at different chord Reynolds numbers (WRLES and WMLES estimates from Choi and Moin [2]).

${Re}_{C}$	WRLES	WMLES	$\mathrm{WMRLES}\;(y_{w}^{+}=1$)	
			r=1.2	r=1.33
$10^{6}$	$5.23\times 10^{7}$	$3.63\times 10^{7}$	$5.22\times 10^{7}$	$4.65\times 10^{7}$
$10^{7}$	$7.76\times 10^{9}$	$8.20\times 10^{8}$	$1.51\times 10^{9}$	$1.28\times 10^{9}$
$10^{8}$	$5.98\times 10^{11}$	$9.09\times 10^{9}$	$2.04\times 10^{10}$	$1.64\times 10^{10}$
$10^{9}$	$4.34\times 10^{13}$	$9.26\times 10^{10}$	$2.44\times 10^{11}$	$1.89\times 10^{11}$

**Table 2:** Ratios of total mesh count (R, Equation (23)) for WMRLES versus WMLES on equivalent base grids, for all channel flow test cases. Grids A through H correspond to the ${Re}_{\tau }=5200$ test case, while Grid I corresponds to the ${Re}_{\tau }=2000$ test case.

Grid	$n_{x}$	$n_{y}$	$n_{z}$	$y_{w}^{+}$	r	$N_{y}$	R
A	8	16	16	162.5	1	16	1
B	8	16	16	1	1.2	39	2.44
C	8	16	16	1	1.33	31	1.94
D	8	16	16	1	1.5	27	1.69
E	8	16	16	2	1.2	35	2.19
F	8	16	16	2	1.33	28	1.75
G	8	16	16	2	1.5	25	1.56
H	16	24	24	1	1.2	45	1.88
I	8	16	16	1	1.2	34	2.13

**Table 3:** List of simulations performed in the present study: column 2 specifies the case Reynolds number; column 3 specifies the wall treatment method; column 4 specifies the mesh as defined in Table 2; column 5 specifies the value of the grid-based mixing length limiter coefficient value (Equation (18)); column 6 specifies the fraction of upwinding used in the low-dissipation hybrid upwind-centered formulation for the convective flux (Equation (33)); column 7 specifies the SGS model used.

Case	Re_τ_	Method	Grid	$C_{d}$	η	SGS
1	5200	WMRLES	B	1	0.2	MILES
2	5200	WMRLES	B	2	0.2	MILES
3	5200	WMRLES	B	3	0.2	MILES
4	5200	WMRLES	B	4	0.2	MILES
5	5200	WMRLES	B	2	0.1	MILES
6	5200	WMRLES	B	2	0.05	MILES
7	5200	WMRLES	B	2	0.05	WALE
8	5200	WMRLES	C	2	0.2	MILES
9	5200	WMRLES	D	2	0.2	MILES
10	5200	WMRLES	E	2	0.2	MILES
11	5200	WMRLES	F	2	0.2	MILES
12	5200	WMRLES	G	2	0.2	MILES
13	5200	WMRLES	H	2	0.05	MILES
14	5200	WMRLES	H	2	0.05	WALE
15	5200	WMLES	A	-	0.2	MILES
16	5200	WMLES	A	-	0.1	MILES
17	5200	WMLES	A	-	0.05	MILES
18	5200	WMLES	A	-	0.05	WALE
19	5200	DHRL	B	-	0.05	MILES
20	5200	DES	B	-	0.05	-
21	5200	IDDES	B	-	0.05	-
22	2000	WMRLES	I	2	0.05	MILES

## Data Availability

The original contributions presented in the study are included in the article, further inquiries can be directed to the corresponding author.
